# Methods of Modifying the Content of Glucosinolates and Their Derivatives in Sprouts and Microgreens During Their Cultivation and Postharvest Handling

**DOI:** 10.1155/ijfo/2133668

**Published:** 2025-01-14

**Authors:** Magdalena Michalczyk

**Affiliations:** Department of Biotechnology and General Technology of Food, Faculty of Food Technology, University of Agriculture in Krakow, Kraków, Poland

## Abstract

Sprouts and microgreens which belong to the Brassicaceae family contain significantly more glucosinolates than mature vegetables, and their composition often differs too. These plant growth stages can be a valuable supplement of the aforementioned compounds in the diet. The content and proportion of individual glucosinolates in sprouts and microgreens can be regulated by modifying the length and temperature of cultivation, the type of light, the use of mineral compounds, elicitation, primming, and cold plasma as well as storage conditions. The way in which sprouts are prepared for consumption affects the yield of glucosinolate hydrolysis. Genetic variation leading to different plant responses to the same factors (e.g., type of light) makes it necessary to conduct detailed studies involving species and variety diversity. Heat stress and the use of cold plasma appear to be fairly universal methods for increasing glucosinolate content. Studies on the use of light at different wavelengths do not provide unequivocal results. Despite experiments on the use of seed soaking solutions (e.g., sulfur and selenium compounds), there are no studies in the available literature on the effects of chemical and thermal seed disinfection methods on the glucosinolate content of the obtained sprouts and microgreens.

## 1. Introduction

Sprouts and microgreens are an attractive food product both for their sensory characteristics and their nutritional and health-promoting value. They are commonly used as an additive to salads and sandwiches or to garnish dishes. Sprouts can also be the base of products such as the fermented drink rejuvelac or bread made from sprouted grain. There is also growing interest in the potential use of sprout flour for making bread and other bakery products [1–3]. Sprouts and microgreens, with a high content of desirable compounds and preparations obtained from them, can be functional foods as well as an ingredient in dietary supplements. Glucosinolates (GSLs), occurring in many plants, are substances of great interest due to their various health-promoting activities. At least 120 of these compounds have been identified, among others, in plants belonging to the families Brassicaceae, Capparaceae, and Caricaceae [4]. According to Vale et al. [5], brassica sprouts are an underutilized source of GSLs. Dietary supplements based on broccoli sprout extract, which are commercially available, are one example of the use of this raw material. There is also a growing interest in microgreens, which represent a slightly longer-growing plant stage than sprouts. Microgreens are usually harvested between 7 and 14–21 days after sowing. Sprouts are consumed whole with roots and seeds; microgreens, in turn, are cut; and roots are left in the ground, not being part of the food product [6].

## 2. GSLs and Factors Affecting Their Hydrolysis in Sprouts

Biosynthesized from amino acids, GSLs consist of an S-*β*-D-glucopyranoside connected to an O-sulfated (Z)-thiohydroximate moiety [7]. In general, they are divided into three groups: aliphatic, aromatic, and indole GSL. The amino acid precursors of the first group are alanine, leucine, isoleucine, methionine, and valine. For aromatic GSLs, these are phenylalanine and tyrosine, while for indole ones, it is tryptophan [8]. GSLs present in undamaged plant tissues are separated from the enzyme myrosinase (*β*-thioglucosidase). After tissue damage, the enzyme comes into contact with the substrate that leads to glucose release. However, for the myrosinase activity, the presence of ascorbic acid is essential. Unstable aglucones (thiohydroxamate-O-sulfonate) are then converted to isothiocyanates (ITCs) and thiocyanates, nitriles, oxazolidine-2-thiones, and epithionitriles. The result of transformations depends on both the GSL substrate and the presence of the specifier proteins (epithiospecifier proteins (ESPs), nitrile-specifier proteins, thiocyanate-forming proteins, and epithiospecific modifying proteins) and the presence of Fe^2+^ as well as pH and temperature [4, 9–12]. ITCs can then be converted to 1,3-oxazolidine-2-thiones and indoles or, by reaction with nucleophilic groups of biomolecules found in cells, to dithiocarbamates, thiocarbamates, and thioureas, which can affect, among others, the functioning of signalling pathways or even the structure disruption of proteins and DNA [11].

Apart from plant tissues, myrosinases are also found in fungus and bacteria, including those present in human and animal gut [4]. However, the effects of GSL conversion by myrosinases of the gut microbiota differ markedly between individuals and often are not very efficient [13]. Fahey et al. [14] report that in various studies, conversion of glucoraphanin (GRA) to sulforaphane (SFN) metabolites ranged from 1% to 40%, while it was 70%–90% conversion to urinary dithiocarbamate metabolites when volunteers obtained SFN. After absorption, ITCs in the human body are metabolized by the mercapturic acid pathway [9].

The products of GSL conversion exhibit many different biological activities. ITCs are involved in protecting plants against pests and microbial attacks. In Japan, plant-derived allyl ITCs are approved for food preservation [15]. Kamal et al. [16] indicate that ITCs such as SFN, phenylethyl isothiocyanate (PEITC), moringin (MG), erucin (ER), and allyl isothiocyanate (AITC) demonstrate various cardio- and neuroprotective activity. According to the works cited by the authors, compounds belonging to ITCs may also have beneficial effects on body weight and many other indicators describing health status including blood glucose level and cholesterol profile. These substances are also supposed to show protective effects against oxidative stress, electrophilic stress, and apoptosis [16]. Due to the anti-inflammatory properties of ITC, opinions are even encountered about the possible advantageous effect of these compounds in the prevention and therapy of endometriosis [17]. Besides their beneficial effects, GSL can also generate problems. The content of progoitrin degrading to goitrin belonging to oxazolidine-2-thiones in rapeseed can result in goitrogenic effects and health problems in livestock fed with rapeseed middlings and cake. This has led to the breeding of this plant varieties with reduced GSL content [10].

Despite the fairly widespread opinion about the beneficial and anticancer effects of the compounds discussed, especially of ITCs, on human health, the results of studies are not quite unequivocal, nor is the recommended dose established [18–21]. Lynn et al. [20] cite the results of epidemiological studies, some of which do not support a beneficial effect of brassica vegetable consumption on lowering the risk of specific gastrointestinal cancers. The authors suggest that the differences between the positive results of the animal studies and the lack of results in the epidemiological studies cited should be considered in the context of the differences between the magnitude of the doses in the two types of study. Lynn et al. [20] also highlight various difficulties in conducting epidemiological studies resulting from not taking into account genetic differences between individuals and the method of collecting information on food intake using food-frequency questionnaires that may cause overestimating vegetable consumption and misclassification as well as not taking into account the effect of different types of vegetable processing prior to consumption The need to consider the relationship between genetic polymorphisms and gut microbiota and the health effects of GSLs and ITCs is also highlighted by Marino et al. [19]. There is also a need to determine appropriate doses of ITCs and GSLs, as both the beneficial effect on health and the possible risks of their use are dose related [18, 21]. The fact that many of the studies are conducted on animal models or cell lines may add a further complication to the evaluation of GSL and their derivatives. On the other hand, many researches indicate beneficial effects of consuming *Brassicaceae* vegetables [12, 16, 22, 23]. Among the GSLs and their derivatives, SFN (1-isothiocyanato-4-(methyl-sulfinyl)butane) formed from GRA, sinigrin-derived allyl ITC, and indole-3 carbinol derived from glucobrassicin, 2-phenylethyl ITC (from gluconasturtiin), benzyl ITC (from glucotropaeolin), and 3,3⁣′-diindolylmethane (product of indole-3-carbinol condensation) have attracted much attention [16, 24, 25]. As was mentioned above, the SFN that belongs to the ITCs undergoes conjugation with glutathione in the body and then, the conjugates hydrolyse and N-acetylcysteine derivatives—mercapturic acids—are formed [14]. Among other things, its anticancer properties related to its capacity to induce Phase II detoxifying enzymes are considered [26]. SFN is formed from GRA. It is not, however, the only conversion product of this GSL. Its hydrolysis can also result in the formation of nitriles, which may be influenced by plant species [13, 27]. According to Singh et al. [12], indole-3-carbinol characterized by low toxicity, may stimulate the production of DNA repair proteins, while preliminary studies suggest its anticancer, antiviral, antifungal, antibacterial, and anti-inflammatory effects.

Kołodziejski et al. [11], who assessed the content and composition of GSL in different plants and stages of growth in radish, Brussels sprouts, savoy cabbage, and white cabbage, report that nearly all GSLs present in other parts of a particular plant were present in sprouts. According to the authors, at the same time, a low efficiency of conversion rate of GSL to ITC and indoles was detected (less than 1%), despite high myrosinase activity at this plant development stage. For the edible parts of analyzed plants, it was more than 70%. De Nicola et al. [28], who compared the percentage conversion of GSLs into ITCs in several 7-day brassica sprouts, found that it was highest in two varieties of radish (96.5% and 90%), followed by Tuscan black kale (68.5%), and was only 18.7% in broccoli. According to the findings of Matusheski, Juvik, and Jeffery [27], the primary GRA hydrolysis product in broccoli crushed at room temperature may be nonbioactive SFN nitrile, whereas the SFN yield observed by the authors was low. After hydrolysis was performed by incubating the homogenate at room temperature, a SFN content of 0.08–0.62 *μ*mol/g fresh weight (fw) and SFN nitrile content of 0.35–1.5 *μ*mol/g fw were noted, with lower SFN content than SFN nitrile content for each of the varieties evaluated in the study. The authors suppose that the ESP, a noncatalytic cofactor of myrosinase, is probably the reason for these proportions of components obtained. ESP is heat-sensitive and requires iron for activity. They observed that heating both broccoli florets and broccoli sprouts to 60°C before crushing them resulted in an increased proportion of SFN in the GRA hydrolysis products. In the case of broccoli, the application of temperatures of 70°C or 100°C led to a significant decrease in the detected hydrolysis products. Interestingly, in the case of sprouts, the effect of even 100°C was much less destructive on the concentration of the obtained hydrolysis products [27]. It appears that heat treatment may primarily degrade the sensory attractiveness and nutrient content of products such as sprouts, while at the same time minimizing the associated microbiological risks. However, the studies cited above indicate a possible positive effect in increasing the availability of GSL derivatives in this product. Another proposal for intensifying SFN formation in broccoli sprouts was presented by Liang et al. [29]. To target hydrolysis for SFN formation, the authors suggest mixing broccoli sprouts with radish, rocket, and rape sprouts. By mixing the sprouts in equal amounts by weight, then juicing them with water, and conducting hydrolysis at room temperature for 1 h, they obtained an approximately twofold increase in SFN level ratio to single broccoli sprouts. In contrast, they assessed mustard sprouts as not useful in this process. The authors emphasized that there was no GRA in the sprouts they added. Fahey et al. [14] report that for processes that the human body is undergoing, it is vital to provide active myrosinase along with GRA in the administered preparations. This leads to increased bioavailability of SFN. The aforementioned authors gave a juice containing vitamin C and prehydrolysed freeze-dried broccoli sprouts, for volunteers. This allowed conversion of GRA to SFN and resulted in about 40% bioavailability. The problems associated with the effective use of SFN from broccoli sprout juice are also highlighted by Bello et al. [30]. During the preparation of broccoli sprout juice, they observed a low, about 25%, yield of SFN and SFN nitrile (compared to the initial content of GRA) and losses of SFN due to spontaneous conversion to SFN-amine or conjugation to glutathione and proteins. Thus, the conclusions of the studies described above do not fully coincide. They indicate the need to develop such methods for the preparation of sprouts/microgreens prior to consumption that will maximize their potential health-promoting properties. It seems particularly interesting to determine the influence of heat treatment parameters prior to subsequent hydrolysis for individual plant species and their growth stage.

## 3. GSL Content of Sprouts and Microgreens Compared to Mature Vegetables

Sprouts and microgreens are a valued product due to high content of bioactive substances, often far exceeding the content of the same components in mature vegetables. For example, according to Hanlon and Barnes [31], radish sprouts had 3.8-fold higher GSL content, 8.2-fold higher ITC content, and 6.9 times higher phenolic content than mature taproots. As reported by Fahey, Zhang, and Talalay [32], the total GSL content in 3-day broccoli sprouts was 22.7 *μ*mol/g fw, while in mature broccoli, it was 3.37 *μ*mol/g fw. The composition of GSL was also different in the both plant stages. In the mature vegetables, 68% comprised indole GSLs; in the sprouts, they accounted for only 3%, while methylsulfinylalkyl GSLs accounted for 20 times more compared to the mature form. According to the authors, this high proportion of methylsulfinylalkyl GSLs, including GRA, in sprouts is crucial when considering the cancer prevention effect of consuming plants containing GSL. Sun et al. [33], who assessed the GSL content of sprouts, rosette leaf, and bolting stem of 27 Chinese kale varieties, found the highest total GSL content in sprouts (58.4–412.4 *μ*mol/g dw (dry weight)), lower in bolting stems (3.4–30.6 *μ*mol/g dw), and lowest in rosette leaves (4.3–17.3 *μ*mol/g dw). The individual varieties differed in the composition of these compounds, including varying contents of glucoiberin and GRA. In the total GSL compounds in the sprouts, the proportion of gluconapin was the highest with regard to the 26 varieties. Higher contents of GSLs than in mature vegetables were also reported by Huang et al. [34] in their study on microgreens. The authors determined 17.2 *μ*mol GSLs/g dw in red cabbage microgreens, while in mature red cabbage, it was 8.3 *μ*mol/g dw. The content of GRA, from which SFN is formed, was 4.8 *μ*mol/g dw and 0.9 *μ*mol/g dw, respectively. There were also more sinigrin (6.1 and 1.5) and progoitrin (6.9 and 2.3). Zou et al. [35], who compared the content of GSLs in choy sum (*Brassica rapa* subsp. *chinensis* var. *parachinensis*) at three growth stages: microgreens (7 days after sowing), seedlings (15 days), and adult (30 days), observed changes in GSL profile during growth. For microgreens, aliphatic GSLs were characteristic, which were replaced by indolic GSLs in seedlings and to aromatic GSL in adult plants. At the same time, the older the plants were, the lower their total GSL content was. The results reported by Sarikamiş, Yildirim, and Alkan [36] do not fully correspond with the aforementioned findings. The authors observed the content of these compounds in seeds, sprouts, and seedlings of white head cabbage and black radish over 20 and 21 days after sowing. They found that for both species, total GSL content was highest in young seedlings with true leaves, compared to seeds and sprouts. In turn, Di Bella et al. [37], comparing three stages of kale growth during the spring season in South Italy, found the highest total glucosinolate (TGSL) content in microgreens, lower in young leaves, and lowest in sprouts. In contrast, broccoli had the highest TGSL content in sprouts. The authors also observed differences in the content and composition of GSLs related to the cultivation season; in autumn, the content of the compounds studied was usually lower than in spring.

The aforementioned examples indicate that the content and composition of the discussed compounds in plants are affected not only by the species, cultivar, or growth stage but also by the growing season if, for example, cultivation is conducted in unheated greenhouse.

## 4. Modification of GSL Content in Sprouts and Microgreens

### 4.1. Cultivation Factors Affecting the Content and Type of GSLs and Their Derivatives in Sprouts and Microgreens

**Figure 1:**
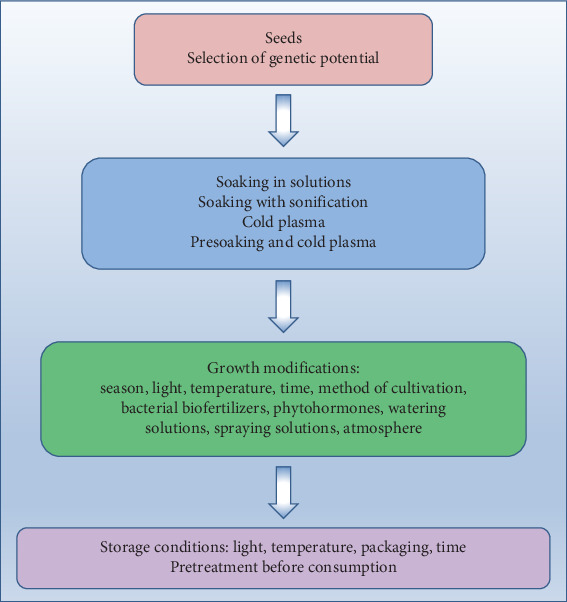
Factors affecting the content of GSLs and their derivatives in sprouts and microgreens.

Both the concentration and type of GSLs present in plants depend on many factors: plant species and cultivar, plant parts, age of plant, and cultivation factors including soil characteristics, climate, growing season, and water availability as well as exposure to pathogens and postharvest climatic conditions [4, 10, 38]. Factors that can influence the characteristics of the obtained sprouts and microgreens, apart from genetic ones, include elicitation, light access and characteristics, and length of growing or temperature (Figure 1). From the point of view of research on the mechanisms determining the content of bioactive compounds in plants, sprouts and microgreens are an attractive raw material due to the short period from sowing to harvest. However, in their case, the results obtained are not always the same as for mature plants [39].

Tilahun et al. [40] assessed the differentiation in contents of essential components in microgreens of various radish cultivars grown in a vertical multilayered growing unit, without substrate. In 10-day microgreens obtained from the seeds of five radish cultivars, there were no statistically significant differences in the total GSL content (from 32.2 to 38.3 *μ*g/g dw); however, statistically significant differences were found in the content of individual GSLs in the plants (e.g., GRA, from 1.5 to 16.2 *μ*g/g dw in different cultivars). The cultivation method can also have an effect on the GSL profile in microgreens. For example, Liu et al. [41], who grew kale and broccoli microgreens under chamber and windowsill conditions, found that for some individual GSLs, there were significant differences in their concentrations between the two cultivation methods. The concentrations of bioactive substances, including sulfur-containing compounds, also changed during successive days of germination. Wang et al. [42] confirmed the well-known fact of the effect of cultivar on GSL content with four cultivars of rocket as an example. The authors also observed varying concentrations and mutual proportions of compounds from this group over 8 days of cultivation, resulting from their synthesis, decomposition, and transformation. They also noted that during germination, the transformation was possible of glucoerucin to GRA which is a precursor of SFN. Myrosinase activity also varied over time and was cultivar dependent. The length of the sprout growth period can significantly affect both the composition and total GSL content. If the aim is to reduce the content of undesirable GSLs, a longer growing period may be considered. For example, Paśko et al. [43] stated a decrease in progoitrin content in rutabaga sprouts after increasing sprouting time from 8 to 10 and 12 days. The decrease in the content of this substance after 10 days compared to 8-day sprouts was 91.5% and after 12 days 97.5%. Progoitrin was the main aliphatic GSL identified by the authors in both seeds and sprouts of rutabaga. Bellostas et al. [44] observed a decrease in the concentration of alkyl GSLs and an increase in the concentration of indol-3-ylmethylglucosinolates over successive sprouting days of white, red, and savoy cabbage; broccoli; and cauliflower. The highest GSL concentration in 4- and 7-day sprouts was determined by the authors in roots, while at the same time, cotyledons had the highest content of alkylthio- and alkylsulfinylglucosinolates.

Among the growth factors, temperature is the factor that can be modified to affect the characteristics of the finished product. However, due to the large amount of heat released during germination, this parameter may not be the easiest to control precisely under industrial conditions. Yang et al. [45] exposed cabbage sprouts from the second day of germination to temperatures of 40°C, 50°C, or 60°C for 20 min every 24 h. The authors observed an increased total GSL content after applying temperatures of 50°C and 60°C. Heat shock affected particularly the content of aliphatic GSLs, which are converted to ITCs, whereas it did not significantly influence the content of aromatic (gluconasturtiin) and indole GSLs. As a result, an increase in myrosinase activity and total phenolic and ascorbic acid accumulation and decrease of ESP activity were also noted. A beneficial effect of elevated cultivation temperature, 30/15°C (day/night) compared to the 22/15°C and 18/12°C regime, on the GSL content of broccoli sprouts was also observed by Valente Pereira et al. [46]. Furthermore, temperature stress in the form of constant low (11.3°C) or high (33.1°C) temperatures increased the GSL content. The highest levels of these compounds the authors determined are in cotyledons and the lowest in roots. In addition, the content of GSL in the sprouts decreased over successive days of cultivation and was highest at the start of harvest (sixth day) and lowest on the 11th day, when the analyses were completed [46]. An increase in TGSL content was also observed after low-temperature stress was applied in growing flat leaf kale sprouts [47]. Control samples were cultured at 21°C, while the others were exposed to 8°C for 24 h or 8°C for 23 h and −8°C for 1 h. The application of 8°C led to a simultaneous increase in the content of aliphatic GSLs and a decrease in the content of indolic GSLs. GRA was detected only in samples exposed to 8°C. However, this temperature significantly reduced root length and yield.

Another option to increase GSL accumulation is the use of elevated CO_2_ [48]. In the experiment reported by the authors, its concentration in the atmosphere was maintained at 620 ppm during 9 days of sprouting. This resulted in both an increase in GRA and myrosinase activity, a reduction in ESPs, and consequently an increase in SFN content and a reduction in SFN nitrile content after the 8-h autolysis of grounded sprouts. However, the resulting changes were also affected by cultivar.

### 4.2. Effect of Storage on the Content and Type of GSLs and Their Derivatives in Sprouts and Microgreens

The content of bioactive compounds available to the consumer is also affected by postharvest storage. It can be generally stated that storage reduces the contents of GSL and their derivatives; however, the results obtained by different authors for individual species are not consistent in details. Li and Zhu [49] observed a decrease in ITC but not in GSL of radish sprouts stored for 12 h at 25°C as well as at 4°C. During the 3-week storage of broccoli, white radish, and kohlrabi sprouts at 4°C, there were no significant changes in the content of individual GSLs. However, such changes concerned rocket sprouts, where a reduction in glucoerucin was observed after 1-week storage and in GRA after 2 weeks [50]. In contrast, Lu et al. [51] when analyzing broccoli microgreens stored for 21 days (4°C, dark) observed a gradual reduction in the two main GSLs determined in the plants: glucoerucin (from 27.5 to 12.6 *μ*mol/g dw in control) and GRA (from 1.6 to 0.7 *μ*mol/g dw in control). Vale et al. [52] in turn examined sprouts of red cabbage, broccoli, Galega kale, and Penca cabbage, which were previously grown with access to light and in the dark and were stored at 4°C for 12 days in the dark. Changes in the GSL content of the stored product were affected by both the plant species and the fact whether it came from a dark or light cultivation. The levels of these compounds were most stable in darkness-grown broccoli and red cabbage sprouts. Galega kale sprouts lost an average (for plants growing in light and in darkness) of 90% of GSLs after 12 days. In the case of broccoli and red cabbage, it was 18% and 39%, respectively.

A few studies have also been conducted on the effect of light during storage of sprouts and microgreens.

The possible use of red and white LEDs (6/24 h, 5 days) on the characteristics of cabbage, radish, and rocket microgreens packed in nonsealed polyethylene terephthalate punnets was evaluated [53]. With regard to white LEDs, 5-day storage resulted in a reduction in 4-methoxyglucobrassicin in cabbage by 42%, glucoraphenin in radish by 5%, and GRA A and glucoerucin in rocket by 3% and 6%. Plants with the access to red light gained 263% (cabbage), 170% (radish), and 29% and 20% (rocket) of mentioned GSLs.

Martínez-Zamora et al. [54] took another attempt to influence the content of the discussed compounds by modifying the lighting during sprout storage. Rocket, radish, and tatsoi sprouts were stored for 5 days at 5°C under white, blue, green, orange, and red LED light. The effect of lighting on SFN content depended on the species and type of light and was often small and not always positive. For rocket sprouts, the largest increase in SFN content was found for blue LED light (16% dw), while for radish and tatsoi, it was red (~10%) and white light (~12.6%), respectively. In turn, Castillejo et al. [55] estimated changes in GSL content in broccoli sprouts, grown in darkness for 9 days and stored at 5°C for 15 days in the dark, under white fluorescent light and white, yellow, and green LEDs. Throughout sprout storage in darkness and under fluorescence light, the total GSL content was decreasing. Storage under LEDs (24 h throughout the shelf-life period) maintained the level of TGSL, but yellow LEDs caused a significant increase in TGSL. Lu et al. [56], who stored broccoli microgreens at 5°C for 21 days, observed a decrease in the content of glucoerucin, GRA, and total GSLs over successive days of analysis. The level of glucoerucin and GRA in control samples on the last day of analyses decreased up to 56%, compared to the initial concentration. In addition, the authors observed that UV-B treatment applied during plant growth lowered the amount of GSL loss (by less than 30%) during later storage. The above-mentioned studies indicate that light access and characteristics are important for the concentration of GSL, not only during the growth period but also during storage. However, the number of papers dealing with this issue is not large, which does not allow definitive conclusions to be drawn. Both the issue of light selection during storage and the influence of different modifications of the growing conditions on the subsequent stability of the bioactive compounds require more extensive research.

### 4.3. Increase of GSL Content in Sprouts and Microgreens by Biofortification

The content of GSLs and their derivatives in sprouts can also be affected by their fortification with minerals. An increase in the GSL content of sprouts was observed both when this compositional modification was the main objective and when the aim was to enrich the product with minerals. Paśko et al. [57], prior to seed sowing, enriched kale sprouts by soaking seeds in solutions containing selenium compounds (Table 1). They found that the addition of a phenylselenyl derivative containing phenylalkylselenyl chain resulted in an extremely strong increase in the concentration of benzyl ITC, phenyl ITC, butyl ITC, and phenethyl ITC and a more than twofold increase in SFN in the final product. According to the authors, other selenium compounds also increased the SFN content and, in most cases, the content of allyl ITC and sinigrin. An increase, compared to the control, was also observed for progoitrin and glucoiberin. In the case of indole ITCs, however, the effect of using additives to enrich seed soaking water was not so clear-cut and both increases and decreases in the concentration of compounds from this group were observed. The authors also note that the organic selenium compounds they used had a much stronger positive effect on the synthesis of sulfur compounds than is reported in the literature for inorganic selenite or selenate and that the method of supplementation may play an important role in its effectiveness. This thesis can be supported by the studies cited below [58, 59], in which the application of SeO_2_ water solution and Na_2_SeO_3_ solution did not significantly increase the content of GSLs (Table 1). However, in another study [60], an increase in the content of some GSL was obtained (Table 1).

In the studies on sprout fortification, a rarely used form of selenium is elemental selenium in the form of a colloidal solution. Vicas et al. [61], who used nanoselenium particles for the biofortification of broccoli sprouts, showed that the content of individual GSLs varied between the samples obtained at different supplementation levels. The presence of selenium reduced the content of glucoerucins and at different doses affected differently the content of GRA. For indolic GSLs, no statistically significant differences were found between the samples.

According to Zhuang et al. [62], due to the numerous health-promoting properties of SFN, there is a great interest in increasing SFN formation in broccoli sprouts. The mineral that can affect the GSL synthesis is also calcium. Among its compounds used were CaCl_2_ and CaSO_4_ [62–64]. The aforementioned authors observed a beneficial effect on the content of GSL or SFN of solutions of these compounds used for irrigation of broccoli sprouts (Table 1). This was associated with enhancement or limitation of the expression of the respective genes. In broccoli sprout cultivation, a variety of substances have been tested as elicitors including NaCl [65]; H_2_O_2_ [66]; methionine, tryptophan, chitosan, salicylic acid, and methyl jasmonate [67]; sucrose, mannitol, NaCl, 1-aminocyclopropane-l-carboxylic acid, and methyl jasmonate [68]; slightly acidic electrolyzed water (EW) [76]; methyl jasmonate, jasmonic acid, and DL-methionine [69]; ZnSO_4_ and melatonin [70]; K_2_SO_4_ [71]. The results indicate that to increase the GSL content, the use of both irrigation solutions and seed priming is very promising (Table 1). For instance, Baenas et al. [69], when using methyl jasmonate, jasmonic acid, and DL-methionine, observed an increase of up to about 100% in the content of TGSL in broccoli and radish sprouts. However, not all work has provided such clear-cut results. Natella et al. [68] showed no significant effect of 1-aminocyclopropane-1-carboxylic acid, salicylic acid, methyl jasmonate, and NaCl on the total GSL content of broccoli sprouts. In contrast, elicitation with mannitol and sucrose proved to be beneficial. Salicylic acid and methyl jasmonate affected the content of some individual GSLs. A similar individual effect on changes in GSL belonging to different classes was observed by Pérez-Balibrea, Moreno, and García-Viguera [67] in 7-day broccoli sprouts. Tryptophan, salicylic acid, and methyl jasmonate increased the content of indole GSLs, while methionine increased levels of aliphatic GSLs. In 3- and 5-day sprouts, no statistically significant effect of the mentioned amino acids was observed.

In the cultivation of microgreens, the application of some compounds including CaCl_2_, [51, 56], CaSO_4_ and NaCl [72], ZnSO_4_ [73], and NaCl [74] was also tested. Preharvest application of CaCl_2_ led to an increase in the content of the discussed compounds in broccoli microgreens [51, 56]. Whereas NaCl solution decreased their content, the combination of NaCl and CaSO_4_ mitigated the decrease, while CaSO_4_ solution alone increased the content of total GSLs [72]. The authors also determined the SFN content obtained after 3-h incubation (37°C) of grounded microgreens. Only the application of NaCl solution increased its content determined under these conditions. The remaining cultivation methods did not significantly affect the level of the mentioned compound.

Among the compounds used for biofortification were also ZnO and *γ*-Fe_2_O_3_ nanoparticles synthesized with the coprecipitation method and functionalized with a *Pseudomonas* species preparation [75]. Assessment of the TGSL content in broccoli microgreens indicated, for example, a clear increase in the content of these substances (162%) after application of bacterium-functionalized ZnO nanoparticles at 250 ppm. The highest GRA content was achieved with uncapped *γ*-Fe_2_O_3_ nanoparticles at 250 ppm in combination with P-solubilizing bacterial biofertilizer (29-fold compared to control), and that for progoitrin with uncapped ZnO nanoparticles at 250 ppm.

The use of both soaking seeds and spraying them with solutions containing minerals or other elicitors appears to be an easy and inexpensive method of influencing the GSL content of sprouts and microgreens. These can also be methods to modify the concentration of individual GSLs, not just their overall amount. However, the difficulties in determining the optimal dose and eventually the combination of additives in solution are also evident from the work collected. This may result not only from the genetic characteristics of the raw material but also, as indicated by works where two or more factors were applied simultaneously, from the influence of other cultivation factors. Interpretation of the results is also made more difficult because of the different experimental designs adopted by the authors: The timing of the start and duration of additive application during cultivation are often different. The times at which the crop is harvested also vary. The selenium example shows that the form in which the mineral is applied also has a significant influence on the result. Advantages were also obtained after the application of, for example, sulfur or calcium compounds. The most equivocal results seem to relate to the use of NaCl, both for the effects on GSL content and yields. An interesting and little studied issue is the effect of the bacterial biofertiliser.

Some papers also reported negative [59, 70, 72, 74] or positive [64, 65, 72] effects of the additives on the fw obtained. Differences in plant weights from control and proper samples can be another reason for problems in comparing results. Since the ingredients used can affect not only the GSL content but also the yield of plants and dry matter content, it seems worth considering methods of expressing GSL content that also show their yield, for example, milligram of GSL/100 g of seed sown.

#### 4.4. Application of Cold Plasma, Electrolyzed Water, and Ultrasound (US) as Novel Elicitors

The use of cold plasma in plant cultivation is a relatively new method. Its effect on plants is explained as a type of stress caused by, among others, generation of reactive oxygen species (ROS) [77]. Attri et al. [78] claim that ROS and nitrogen species formed under the influence of atmospheric pressure plasma may pass through the seed coat reaching the endosperm or embryo. The authors found that extending the time of plasma treatment from 3 to 9 or 30 min adversely affected germination of radish seeds (10% or 20%–22% reduction compared to control). Several studies have evaluated the applicability of cold plasma treatment on ITC or GSL content in microgreens and sprouts [77, 79–81]. In all of these studies, there was an increase in the content of the ITC or GSL in microgreens and sprouts obtained from cold plasma–treated seeds, compared to controls (Table 2).

Matra et al. [80] treated previously overnight presoaked mustard green seeds with cold plasma. There were no differences in the microgreens obtained from such seeds in terms of indicators such as germination, length of stem, fw, and dw, although the total ITC content was higher.

The use of EW in the context of sprouts initially attracted interest mainly as a method of improving microbiological safety. There are various types of EW. HOCl- and Cl_2_-containing slightly acidic electrolyzed water (SAEW) with a pH of 5–6.5 is produced when a 2%–6% solution of HCl is placed in an electrolytic cell not divided by a membrane [86]. Electrolyzed oxidizing water, collected at the anode (pH 2–3), is obtained by electrolysis of NaCL solution in a cell in which the anode and cathode are separated by a diaphragm [87, 88]. In this case, HOCl and also HCL, hypochlorite ions (¯OCl), and Cl_2_ are also formed [89]. Basic EW (pH 10–13) can be collected at cathode [88]. Using the first two types of EW discussed, the possibility of reducing microorganisms belonging to Enterobacteriaceae on seeds intended for sprouts [90] and the prospects of inactivating *Salmonella* and *Escherichia coli* O157:H7 on seeds and sprouts [91, 92], among others, have been evaluated. However, the possibility of affecting the content of bioactive compounds in plants with EW is also of interest. The effect of this elicitor on the GSL content of sprouts has been studied in several papers, and an increase in total GSL content under its influence has been observed (Table 2). Noteworthy is the use of a modification involving electrolysis of the CaCl_2_–HCl solution, which led to the formation of Ca^2+^ ions in the seed soaking solution in addition to HOCl, ¯OCl, Cl¯, and H_2_O_2_ [82]. This increased both the calcium content and the total GSL content in the sprouts compared to those treated with tap water [82, 83]. In addition, there was an increase in the content of GRA, which was the predominant GSL. These differences were observed on all cultivation days (2, 4, 6, and 8 days). For the remaining aliphatic (glucoerucin and glucoalyssin) and indole GSLs, the differences were often not statistically significant [82].

Until now, a relatively uncommon technique for sprout modification has been the application of US. Chen et al. [93], after applying US to soybean seeds, observed, for example, cracks and holes in the seed coat, which accelerated water absorption and, in addition, resulted in shorter sprouting time, enhancing also the activities of certain enzymes. Yang et al. [94] showed increased germination rate and gamma-aminobutyric acid content in sprouts of the same plant. However, this technique did not allow Martínez-Zamora et al. [85] to obtain increased GSL content in rocket sprouts. The sprouts from US-treated seeds at harvest had significantly lower Dsf-GRA, siringin, Dsf-glucobrassicin, and Dsf-neoglucobrassicin contents than the control.

#### 4.5. Effect of Light on the GSL Content in Sprouts and Microgreens

Sprouts and microgreens are often cultured indoors, which provides great opportunities for light control. The use of LEDs is the object of an increasing number of studies due to energy efficiency, limited heat release, and the possibility to select precisely spectra composition and selection of a narrow waveband [95]. Light is of fundamental importance for plant growth and characteristics. From the studies described, however, it is often difficult to draw clear conclusions about its effect on GSL accumulation. For example, it is difficult to establish the role played by red and blue light (Table 3). Both of these wavelength ranges (blue 400–500 nm and red 600–700 nm) are often used in research as they are the most effectively utilized for plant photosynthesis [112] and affect morphogenesis [113], as well as are regulators of secondary metabolite biosynthesis[114]. For instance, Chen et al. [96] found a higher GSL content in Chinese kale sprouts grown under blue LED light than under red LED light with simultaneous abundant GSL biosynthetic gene transcripts under red light. According to the authors, the phenomenon described is therefore due to the degradation of the compounds investigated. At the same time, they noted that different red/blue light ratios did not affect the content of indolic GSLs, while blue light increased the amount of aliphatic GSLs compared to white and red lights. Yang et al. [97] also observed an increase in GRA content in broccoli sprouts grown under blue LEDs compared to red LEDs. As reported by Lee et al. [98], kale microgreens illuminated with white and blue LED light contained slightly more GSLs; red LEDs were slightly less beneficial in this respect. On the other hand, Wang et al. [8], who compared the results obtained in white, red, blue, and 75%red + 25%blue LED lights, found that red light promoted GSL biosynthesis and SFN accumulation in broccoli seedlings. They observed that red light could induce SOT18 (GSL biosynthetic pathway gene) expression and upregulation of CYP79B2, CYP79B3, and CYP83B1 (genes involved in secondary metabolism and detoxification), while blue light led to downregulation of SOT18 and low level of methionine content as well as inhibition of aliphatic GSL biosynthesis. Demir, Sarikamiş, and Seyrek [99] noted that blue LED light increased the content of TGSL and GRA in broccoli microgreens. As regards cabbage microgreens, white, blue, red + far red, blue + far red, red + blue, and red + blue + far red combinations resulted in comparable and high contents of total GSL and GRA. With regard to radish microgreens, the application of blue + far red, red + blue, and red + blue + far red lights markedly reduced the content of TGSL compared to white, red, blue, far red, and red + far red lights. The highest glucoraphenin content had radish cultivated under blue and red + far red lights. Guijarro-Real et al. [100] evaluated the ability of using white, blue, and red LED lights, to modify GSL content in sprouts of *Sinapsis alba*, *Brassica nigra*, and *Brassica carinata*. A comparison of the effect of light on *Brassica carinata* shows a definite advantage of red light (48 mg/100 g fw aliphatic GSLs and 33 mg/100 g fw indolic GSLs) over blue (13 and 16) and white (5 and 7) lights. In the case of *Brassica nigra* sprouts, the most apparent beneficial effect was of blue light (39 and 30 mg/100 g fw). White and red lights enabled the following amounts to be obtained: 3 and 4 mg/100 g fw and 6 and 7 mg/100 g fw, respectively. According to some papers [101, 102], no significant differences were found in the GSL content of plants growing under different light conditions. Differences related to cultivar were more important [102]. The examples cited above indicate the individual character of lighting-induced changes. Similar findings on the effect of light on uptake of nutrients in plants are presented by Trivellini et al. [113]. Based on a review of the studies, the authors indicate that the possible light-induced increase in the uptake of a particular nutrient by a plant depends on the type of nutrient, the plant species, and the ratio of red to blue light [113]. Plants take up nutrients through the root system. Light signals reaching leaves and stems, by modulating pathways involving, among others, phytohormones like auxin and brassinosteroids, Ca^2+^, sucrose, proteins, and microRNA, can influence nutrient uptake by roots as well as their usage [113, 115]. At the same time, as can be seen from Table 1, the certain minerals absorbed by the plant can influence the accumulation of GSLs. The phenomena described show the complexity and interplay of factors that can determine the GSL content of sprouts and microgreens. Murthy et al. [114] note that despite the general knowledge that different photoreceptors in plants detect and absorb light signals of a specific wavelength and transmit the signal through a cascade, resulting in modifications of gene expression and metabolic and physiological responses, the complete mechanism of these processes has not been studied and is not fully understood.

Additional complications in the search for a reproducible pattern of light effects may also be due to differences in the assumptions of the experiments: the starting moment, length of the exposure period, photoperiod, intensity of light, and other accompanying cultivation factors. Results concerning the effect of photoperiod alone on GSL accumulation are similarly inconclusive [96, 103].

Trials were also carried out on the application of UV-A, UV-B, and UV-C light during the growth of sprouts [106, 107, 110] observing an increase in the content of GSL depending on the type and intensity of light and the moment the plants were harvested. A beneficial effect on the GSL content in radish and broccoli sprouts was also observed after the application of UV-B or UV-C lighting as a postharvest abiotic stress [108]. In the case of radish sprouts in particular, this treatment increased the GRA content. The simultaneous application of both UV-B and UV-C did not provide additional advantages compared to using each lighting separately. When using UV light, it is important to select an appropriate short exposure as it can have a detrimental effect on plants, degrading their quality [114].

In addition to health effects, the sensory attributes of food products are decisive for consumers. Publications on the possibility of modifying the composition of GSLs usually do not pay attention to their effects on sensory characteristics. Qian et al. [105] highlight that, in addition to health effects, modifying the GSL profile may also be important for the product palatability. The Chinese kale sprouts they cultured using blue LEDs were characterized by the reduced gluconapin content in shoots and increased GRA level in roots, compared to those grown in the dark and under white and red lights. The authors point out that gluconapin after conversion to ITC is a major determinant of bitter taste of Chinese kale sprouts. For all the lighting types mentioned, the total GSL content in root was not significantly different. In contrast, this indicator for the shoots growing in blue light was significantly lower than for the remaining sprout samples.

## 5. Conclusions

The sprouts and microgreens of cruciferous vegetables are raw materials exceptionally rich in GSLs. Both the content and composition of the group of these compounds can be modified by appropriate growing conditions. Factors that allowed their content to be increased included heat stress, EW, or application of cold plasma. Beneficial results were also obtained by using certain solutions, for example, containing organic forms of selenium or CaSO_4_, for seed soaking or sprout spraying. Often, however, the effect of the application of a particular agent depended not only on the concentration of the solution and the plant variety but also on the day of cultivation and the type of GSL analyzed. Studies on the role of light, especially red and blue light, also gave no clear answers. The results depended, among others, on the plant species. In contrast, more unambiguous and promising results were observed during experiments involving UV light, especially UV-B. On the other hand, extension of the cultivation time may lead to a reduction in GSL content, which in some cases may be a desirable modification of the plant composition. One area that has not been investigated so far is the effect of chemical (e.g., sodium hypochlorite solutions) and thermal seed disinfection methods on the GSL content in resulting products. Furthermore, aspects of the influence of the storage conditions of sprouts/microgreens and their preparation for consumption on their health-promoting potential are not sufficiently clarified. Another issue very rarely addressed in research is the effect of compositional modification and GSL content on the sensory characteristics of sprouts and microgreens. Since, in many cases, studies on the influence of a given cultivation factor do not allow clear conclusions to be drawn, perhaps, a more practical approach than the search for general regularities would be to select specific varieties and specific conditions for their cultivation and postharvest treatment that would enable products with distinctive health-promoting properties to be obtained.

The overview of publications also indicates that the ways of expressing the GSL content per fresh or dry matter do not take into account possible yield differences resulting from the modification applied. Perhaps, it would be useful to use an additional indicator, for example, expressing the weight of GSLs per weight or number of seeds used in the experiment.

## Figures and Tables

**Table 1:** Effect of biofortification on content of glucosinolates in sprouts and microgreens.

**Product**	**Treatment conditions**	**Result**	**Reference**
7-day kale sprouts	Immersion for 3-h seeds in organic selenium compound solutions (15 mg/L)	↑ SFN, ↑ TGSL	[57]
7-day white cabbage, broccoli, mustard sprouts	Sprinkling SeO_2_ (10 mg/L) solution	Glucosinolate content and composition not considerably affected	[58]
8-day broccoli microgreens	Spraying (100 *μ*mol/L Na_2_SeO_3_ solution) Spraying and UV-A (40 *μ*mol/m^2^/s)	No effect on TAGSL, TIGSL, ↓ fresh weight increase in aliphatic but no indol glucosinolates	[59]
Kale sprouts analysis (1–7) day after sowing	Soaking of seeds (5 h) in solutions of Na_2_SeO_3_ (10, 20, and 40 mg/L of Se); MeJa (25, 50, and 100 *μ*M); K_2_SO_4_ (30, 60, and 120 mg/L of S)	Effect depending on plant variety, day of analysis, concentration of solution, and type of glucosylate. Na_2_SeO_3_ ↑ 4-MGBS, glucoiberin, glucobrassicin. K_2_SO_4_ ↑ GRA, glucoeurocin, ↑ 4-MGBS, glucoiberin	[60]
9-day broccoli sprouts	Sprinkled with selenium nanoparticles in colloidal solution (10, 50, and 100 ppm)	Effect depending on dose and type of glucosinolate, usually negligible or negative	[61]
5- and 8-day broccoli sprouts	Sprayed: 2.5 mM ZnSO_4_ (control); 10 mM CaCl_2_; 5 mM Ca^2+^ chelator (EGTA); 5 mM Ca^2+^ channel blocker (verapamil)	ZnSO_4_ + CaCl_2_ or ZnSO_4_ + verapamil ↑ GRA, glucoerucin, gluconapin and SFN ZnSO_4_ + EGTA ↓ GRA, glucoerucin, gluconapin, SFN	[62]
9-day broccoli sprouts	Irrigation with CaCl_2_ solutions (5, 10, and 15 mmol/L)	At concentrations of 5 and 10 mmol/L ↑ individual and total GSL (about 1.7-fold). In the dose of 15 mmol/L ↔	[63]
3-, 6-, and 9 -day broccoli sprouts, 9-day stored at 4°C up to 15 days	Spraying with 10 mM CaSO_4_ and 0.01% Tween solution	6th and 9th day ↑ GSL, SFN; during storage, CaSO_4_-treated sprouts lost less of both GSL and SFN than the control	[64]
Broccoli sprouts analyzed on the 3rd, 5th, and 7th day	Sprouts were grown on 0.5% agar with added 20, 40, 60, 80, and 100 mmol/L of NaCl in a culture flask	↔ or ↓ TGSL. In 7-day sprouts, each salt concentration reduced GRA content but 100 mmol ↑ SFN, ↔ SFN in the remaining concentrations	[65]
7-day broccoli sprouts	Spraying H_2_O_2_ (200, 500, and 1000 mM solution)	The higher the H_2_O_2_ concentration, the greater the concentration of GSL, but at 1000 mM morphological changes in sprouts	[66]
Broccoli sprouts, analyses on the 3rd, 5th, and 7th day	Methionine, tryptophan, chitosan, SA, and MeJa were sprayed at different concentrations	Effect depending on the agent, its concentration and day of measurement, and the type of glucosinolate	[67]
5-day broccoli sprouts	Sucrose, mannitol, NaCl, 1-aminocyclopropane-l-carboxylic acid, SA, and MeJa in different concentrations	Mannitol 176 mM and sucrose 176 and 88 mM ↑ TGL; remaining ↔ TGSL	[68]
8-day broccoli and radish sprouts	MeJa, jasmonic acid, and DL-methionine different concentrations of solutions for priming (24 h) and/or for exogenous spraying	In most cases, ↑ in the content of all GSLs are found. In the case of radish, more often than in the case of broccoli, the increases are verified as statistically insignificant	[69]
Broccoli sprouts, examined on Days 4 and 6	4 mM ZnSO_4_ or/and 10 *μ*M melatonin, for irrigation	ZnSO_4_ and ZnSO_4_ + melatonine ↑ SFN, ITC, TGSL, and myrosinase activity. Melatonin ↑ ITC	[70]
Broccoli sprouts examined on Days 3, 6, 9, and 12 after sowing	K_2_SO_4_ (15, 30, and 60 mg/L of sulfur) for soaking of seeds overnight and irrigation during germination	↑ glucosinolate content compared to control evident only 9 and 12 days	[71]
9-day broccoli microgreens	Watered with 80 mmol/L NaCl; 1.39 mmol/L CaSO_4_; 80 mmol/L NaCl + 1.39 mmol/L CaSO_4_	CaSO_4_ ↑ TGSL, the remaining caused their ↓. NaCl caused ↑ SFN and remaining ↔	[72]
Chinese kale microgreens harvested 4, 5, 6, and 7 days of growing	Spraying with ZnSO_4_ solutions (2, 4, 6, 12, and 18 mM)	18 mM ↑ TGSL in 7-day sprouts compared to control, 2 and 4 mM ↓ TGSL, and 6 and 12 mM ↔ TGSL	[73]
14-day microgreens of *Brassica carinata* L.	100 mM NaCl in nutrient solution + fluorescent, blue, red light	Effect depending on type of light and type of glucosinolate. ↑ sinigrin, other GSLs were affected in different ways	[74]
12-day broccoli microgreens	Plant growth–promoting (PGP) bacterial consortia; ZnO (77 nm) and *γ*-Fe_2_O_3_ (68 nm) nanoparticles functionalized with *Pseudomonas* and others	Largest ↑ TGSL for bacteria-functionalized Zn nanoparticles at 250 ppm, followed by uncapped ZnO nanoparticles at 250 ppm and uncapped *γ*-Fe_2_O_3_ nanoparticles at 250 ppm. Largest ↓ TGSL for mineral *γ*-Fe_2_O_3_nanoparticle precursor + P − solubilizing bacterial biofertilizer	[75]

*Note:* ↑, increase in content; ↓, decrease in content; ↔, result not significantly different from control.

Abbreviations: 4-MGBS, 4-methoxy-glucobrassicin; GRA, glucoraphanin; MeJa, methyl jasmonate; SA, salicylic acid; TAGSL, total aliphatic glucosinolates; TIGSL, total indolic glucosinolates.

**Table 2:** Effect of cold plasma, electrolyzed water, and ultrasound on isothiocyanate and glucosinolate content of sprouts and microgreens.

**Product**	**Treatment conditions**	**Result**	**Reference**
7-day Thai rat-tailed radish microgreens	Cold plasma (21 kV, 5 min) and/or spraying with 160 mmol/L NaCl or 10 mmol/L CaCl_2_ or 176 mmol/L sucrose	Highest content of total ITC for CaCl_2_ + plasma, followed by CaCl_2,_ plasma; ↔ ITC for sucrose; ↓ ITC for NaCl	[77]
7-day mustard green microgreens	Cold plasma (21 or 23 kV for 5 min)	↑ total isothiocyanates	[79]
7-day mustard green microgreens	Plasma treatment (21 kV for 5 min) after presoaking overnight	↑ total isothiocyanates	[80]
5-day broccoli sprouts	Cold plasma (30 kV; 1, 2, and 3 min)	↑ glucosinolates and ↑ myrosinase activity; after 2-min treatment with cold plasma ↑ also sulforaphane	[81]
Broccoli sprouts	CaCl_2_-HCl EW, 5 mM CaCl_2_, ACC 10 mg/L, pH 5.5, seeds soaked for 3 h	↑ total glucosinolates and GRA	[82]
Broccoli sprouts	CaCl_2_-HCl EW, 5 mM CaCl_2_, ACC 10 mg/L, pH 5.5, seeds soaked	↑ total glucosinolates	[83]
Broccoli sprouts	SAEW, ACC from 10.6 to 50.3 mg/L, seeds soaked for 3 h	↑ GRA, GER, PROG, GBS, 4MOGBS, and total glucosinolates; ↓ 4OHGBS	[76]
Wild turnip sprouts Broccoli sprouts	EW was exogenous spraying on the cotyledons from Day 3 to Day 10 of sprouting	↑ TGSL and HGB and NGB ↑ TGSL, HGB, MGB, and NGB; ↓GBS	[84]
7-day rocket sprouts	Ultrasound treated seeds in water (30 min, 35 kHz)	30.6 g/kg TGSL for control 25.2 g/kg for US	[85]

*Note:* ↑, increase in content; ↓, decrease in content; ↔, result not significantly different from control.

Abbreviations: 4MOGBS, 4-methoxyglucobrassicin; 4OHGBS, 4-hydroxyglucobrassicin; ACC, available chlorine concentration; GBS, glucobrassicin; GER, glucoerucin; GRA, glucoraphanin; HGB, hydroxyglucobrassicin; MGB, methoxyglucobrassicin; NGB, neoglucobrassicin; PROG, progoitrin.

**Table 3:** Effect of light type on glucosinolate content in sprouts and microgreens.

**Product**	**Treatment conditions**	**Result**	**Reference**
Chinese kale sprouts, analyses on Days 2, 3, 6, and 9	Different photoperiods light (h)/dark (h): 0/24, 8/16, 12/12, and 16/8; white or red (R) + blue (B) LED light at different ratios: 10:0, 8:2, 5:5, 2:8, and 0:10	Greater GSL content under blue light. Effect of photoperiod on GSL content varied on successive days of growth	[96]
5-day broccoli sprouts	Blue, red, blue + red, red + UV − A LED lights, temperature 15°C, 19°C, and 23°C	Blue lights ↑ GRA, red light ↓GRA	[97]
10-day kale microgreens	White, red, and blue LED lights	White and blue light resulted in slightly higher levels of progoitrin, GRA, sinigrin, and glucobrassicanapin compared to red light	[98]
13-day broccoli and cabbage, 10-day radish microgreens	Red, blue, and far-red LED lights, individually and combined	Effect depending on plant species and type of glucosinolate	[99]
7-day mustard sprouts: *Sinapis alba*, *Brassica nigra*, and *Brassica carinata*	Blue or red LED for 3-day-old sprouts (16/8 h)	The effect on GSL depended on species. *Sinapsis alba* was the least sensitive to treatment. The TGSL in black mustard was affected best by blue light, while in the case of Ethiopian mustard by red light	[100]
14-day canola sprouts	White, blue, red, and blue + red LED lights	The TGSL under white, blue, and red light was similar; under blue + red, it was lower	[101]
White- and yellow-flowering rapeseed sprouts and seedlings	Blue and red LED lights (31.7%blue + 66.3%red or 14.8%blue + 81.3%red)	The effect depended on cultivar and stage of growth and type of glucosinolate	[102]
8-day cabbage and Chinese kale microgreens	Red : blue : green LEDs = 1 : 1 : 1; different PPFDs and photoperiods (2, 14, 16, 18, or 20 h·day^−1^)	For cabbage, no effect of photon flux density on TGSL content. As for cabbage, the longer the photoperiod, the more the TGSL. For Chinese kale, 16 h resulted in the least GSL concentration	[103]
8 varieties of 15–16-day-old *Brassica* microgreens	Amber, blue, and red LEDs in mixtures	Effect depended on varieties, type of glucosinolate, and type of light mixture. No unified pattern was found	[104]
7-day Chinese kale sprouts	White, red, and blue LEDs	Blue light ↓ gluconapin in shoots and ↑glucoraphanin in roots	[105]
8-day-old broccoli sprouts	UV-A or UV-B radiation for 120 min (on the 7th day after sowing) alone or in combination with a 25 *μ*M MeJa solution spraying	UV-B caused an increase in the glucosinolate content, while UV-A alone was not sufficient. UV − A/UV − B + MeJa led to an increase in the GSL content	[106]
7- and 8-day broccoli sprouts	Single UV-A or UV-B exposure for 120 min (7-day-old sprouts), harvested 2 and 24 h after lighting	After 24 h, ↑ glucosinolates; after 2 h with one exception ↑ glucosinolates	[107]
10-day broccoli and radish sprouts analyzed up to the 10th day of storage	UV-B and/or UV-C treatments were applied after harvesting and after 24 h on the first day of the shelf-life period	UV-B or UV-C alone has had a beneficial effect on TGSL; UV-B and UV-C gave a weaker effect	[108]
8-day broccoli microgreens	Red : green : blue = 1 : 1 : 1 LED lights, different PPFDs	TAGSL and TGSL increased with light intensity although the differences were not always significant	[109]
Black kale sprouts analyzed on 3, 5, 7, and 10 days of growing	UV-B in total 5, 10, and 15 kJ m^−2^ applied on the 3rd, 5th, 7th, and 10th sprouting day	UV-B ↑ TGSL content	[110]
6-day Chinese kale sprouts	Preharvest red LED irradiation (24 h)	Delayed degradation of aliphatic, indole, and total glucosinolates during 3-day postharvest storage at 20°C	[111]

*Note:* ↑, increase in content; ↓, decrease in content; ↔, result not significantly different from control.

Abbreviations: GRA, glucoraphanin; MeJa, methyl jasmonate; PPFD, photosynthetic photon flux density; TAGSL, total aliphatic glucosinolates.

## Data Availability

The author has nothing to report.
